# An unusual case of yawning during sleep associated with sleep bruxism and obstructive sleep apnea–a case report

**DOI:** 10.3389/fmed.2025.1596512

**Published:** 2025-06-26

**Authors:** Monika Michałek, Karol Marschollek, Wiktor Kuliczkowski, Marta Waliszewska-Prosół, Anna Wojakowska, Katarzyna Madziarska, Helena Martynowicz

**Affiliations:** ^1^Department of Diabetology, Hypertension and Internal Diseases, Wroclaw Medical University, Wroclaw, Poland; ^2^Department of Neurology, Wroclaw Medical University, Wroclaw, Poland; ^3^Clinical Department of Cardiology, Department of Cardiology, Institute of Heart Diseases, Faculty of Medicine, Wroclaw Medical University, Wroclaw, Poland

**Keywords:** yawning, sleep bruxism, obstructive sleep apnea, polysomnography, case report

## Abstract

The current case report presents an unusual coincidence of yawning and sleep bruxism during sleep in a patient with obstructive sleep apnea (OSA). Both conditions were previously defined in the literature as challenging, multidisciplinary problems with complex and little-known pathogenesis. A 71-year-old man with a history of coronary artery disease underwent video-polysomnography (vPSG) due to the suspicion of OSA. vPSG results confirmed severe obstructive sleep apnea, sleep bruxism, and frequent episodes of yawning during sleep. Therapeutic intervention included positive airway pressure therapy and resulted in the resolution of apneic events. Interestingly, PAP titration also reduced the frequency of episodes of sleep bruxism and yawning. Results of the current case report suggest a temporal relationship between desaturation and yawning episodes, thus indicating the hypoxic basis for this behavior. Resolution of yawning after PAP therapy appears consistent with this theory. An instrumental approach to OSA diagnosis supplemented by video-recording allowed the diagnosis of the unusual presence of yawning during sleep.

## 1 Introduction

This is a case report of a patient diagnosed with obstructive sleep apnea (OSA) and sleep bruxism (SB) with concomitant yawning during sleep. To our best knowledge, no previous studies have shown the temporal relationship between yawning and SB. Both behaviors, SB and yawning, are considered sleep-related, and both are still insufficiently explored. SB affects approximately 21% of the global population, with some discrepancies depending on the diagnostic criteria and assessment methods (1). In contrast, yawning during sleep is a very rare physiological behavior observed across various sleep stages; however, specific prevalence data in the general population remain limited.

Several theories have been proposed to understand the role of yawning, some focusing on thermoregulation, others on blood oxygenation, while the implications of SB remain vague.

Although yawning is a widely observed phenomenon, its role is still unclear. Several assumptions were reported in the literature to address this issue. Over time, authors suggest that yawning is linked with the thermoregulatory process, brain cooling, blood oxygenation, arousal mechanism and alertness, non-verbal communication or social aspects, and stress (2–4).

The most important theoretical and conceptual frameworks for sleep bruxism indicate that this phenomenon has a wide and relevant impact on oral and general health. Sleep bruxism, which is defined as repetitive masticatory muscle activity during sleep (5, 6), has been previously linked with headache (7), obstructive sleep apnea (8), perceived stress (9), and masticatory muscle pain (10). Its multifactorial etiology remains insufficiently explored, but recent studies highlighted the role of genetic predisposition (11) and environmental and psychosocial risk factors.

Our case report aims at showing yawning and sleep bruxism as challenging, multidisciplinary problems with multifactorial, complex, and little-known pathogenesis.

## 2 Case description

### 2.1 Patient information

A 71-year-old man suspected of having OSA was admitted to the Department of Internal Medicine, Occupational Diseases, Hypertension, and Clinical Oncology at the Wroclaw Medical University in Poland to undergo polysomnography. The patient was initially referred to the Sleep Laboratory from the Cardiology Unit. During the medical interview, typical symptoms of OSA, including loud snoring, witnessed apneas during sleep, and frequent awakenings from sleep, were reported by the patient.

### 2.2 Medical history

The patient’s medical history included hospitalization due to acute coronary syndrome (NSTEMI) about 1 year before admission to the Sleep Laboratory. He underwent percutaneous cardiovascular intervention, including angioplasty with drug-eluting stent implantation (PCI RCA + DES). Other comorbidities involved arterial hypertension, prediabetic fasting blood glucose, hypercholesterolemia, benign prostate hyperplasia, and nicotinism in the past. Currently applied drugs involve acetylsalicylic acid 75 mg, pantoprazole 20 mg, ramipril 5 mg, and atorvastatin 80 mg.

### 2.3 Informed consent

The patient signed a consent form and participated in the interview, physical, and instrumental examination voluntarily. The study was approved by the local Ethics Committee (no. KB-523/2021) and was conducted following the principles of the Declaration of Helsinki.

### 2.4 Clinical findings

The patient’s physical examination was within normal limits. The estimated BMI was 26.42 kg/m^2^. Vital signs at admission were as follows: blood pressure 121/75 mmHg, heart rate 68/min regular, respiratory rate 14/min, and body temperature 36.9°C. Patient was well-developed, well-nourished, appeared to be of stated age, alert, and fully oriented. Recent and remote memory were intact. He had good insight and cognitive function, without aphasia, dysarthria, or hoarseness. Lips had normal color, without lesions. Teeth were present, and dental hygiene was moderate. Gingiva and mucous membranes were pink without bleeding, lesions, or signs of inflammation. The tongue was of normal size and papillated with midline protrusion. Tonsils were not enlarged, the palate was elevated symmetrically, and the pharyngeal reflex was present.

### 2.5 Timeline

**Table:** 

Date	Event
29 March 2023	Diagnostic video-polysomnography
30 March 2023	severe OSA diagnosis
18 April 2023	In-lab PAP titration
27 November 2023	outpatient clinic control of treatment

**Figure 1:**
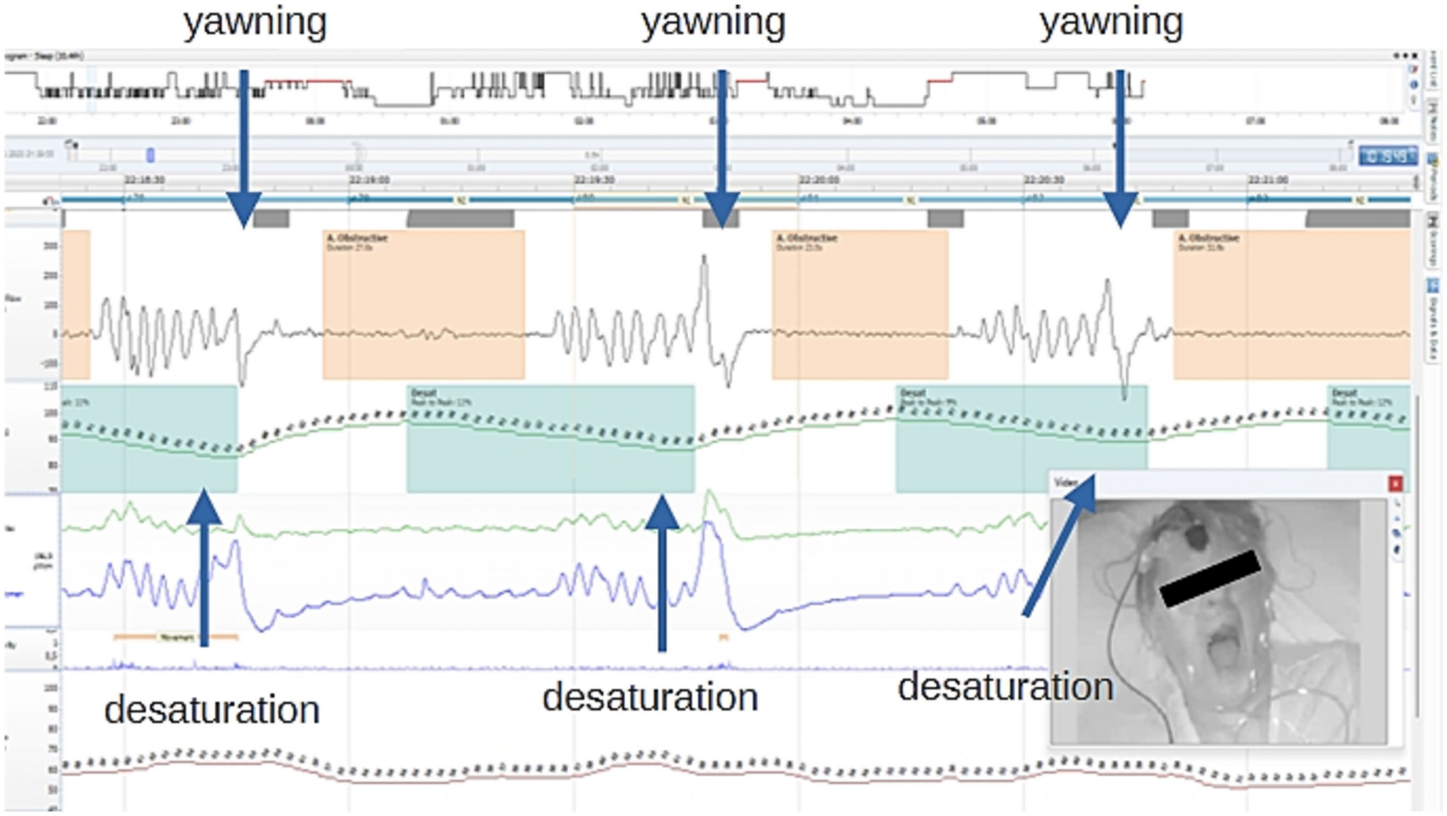
Representative screenshot from the polysomnography recording illustrates the temporal association between yawning episodes and desaturation.

The patient underwent diagnostic video-polysomnography on 29 March 2023, which confirmed a diagnosis of severe OSA. This was followed by an in-laboratory PAP titration on 18 April 2023. After initiating autoPAP therapy, a follow-up evaluation was conducted on 27 November 2023. Although polysomnographic parameters had improved, the patient reported low adherence and treatment-related discomfort, ultimately leading to discontinuation of therapy (see Figure 1).

## 3 Diagnostic assessment

### 3.1 Laboratory findings

Blood samples were obtained by venipuncture after 12 h of overnight fast. Laboratory tests were made in the Hospital’s Main Laboratory. Laboratory testing revealed mild anemia with a hemoglobin concentration of 13.5 g/dL and elevated NT-proBNP 177.4 pg./mL. Other standard blood tests were within normal limits: electrolytes and inflammatory markers such as C-reactive protein, leukocytes, and fibrinogen were at normal levels.

### 3.2 Polysomnographic evaluation

Two overnight vPSGs were performed at a 3-week interval. The first PSG examination was performed to diagnose the patient’s disorder; the second PSG was applied with concomitant positive airway pressure titration. PSG recordings were analyzed by a physician, a certified polysomnography specialist. A Nox-A1 device was used twice (Nox Medical, Reykjavík, Iceland) for vPSG with audio recordings. PSG data was recorded and analyzed thoroughly, including analysis of audio and video recordings, yawning episodes, sleep latency, total sleep time, arousal index, sleep efficiency (%); the ratio of N1, N2, N3, and REM (rapid eye movement) sleep; analysis of movements and sleep position; jaw muscles’ electromyography (EMG) recordings, respiratory events recorded by a nasal pressure transducer, and arterial oxygen saturation (SpO_2_). vPSG recordings were scored with the standard criteria of the American Academy of Sleep Medicine (AASM) Task Force. Apneas were diagnosed when there was a cessation of airflow for ≥10 s, while hypopnea was confirmed when there was a reduction in the amplitude of breathing by ≥30% for ≥10 s with a ≥ 3% desaturation or related to an arousal. SB events were assessed based on EMG recordings from bilateral masseter muscle activity supplemented by audio and video. Bruxism episodes were detected when the increase in the electromyographic amplitude was at least twice that of the background EMG. Electromyographic bursts within 3 s were considered to be part of the same episode. Subsequently, the number of bruxism events per hour of sleep was determined as the bruxism episode index (BEI). According to the scoring of rhythmic masticatory muscle activity (RMMA), a phasic episode included three or more bursts lasting 0.5–2 s, and a tonic episode included a single burst lasting longer than 2 s. The increase in electromyography had to have an amplitude at least twice that of the background electromyographic activity (12).

#### 3.2.1 First polysomnographic examination

During the first diagnostic night, we performed vPSG and observed 34 episodes of yawning during sleep, which were linked to phasic bruxism episodes. Some of them were present in clusters, lasting from 4.6 s to a maximum of 31.2 s, and observed and heard in the video and audio recordings. Yawning episodes were observed before, in the middle, or at the end of phasic bruxism episodes, mostly in N1 and N2 sleep stages, followed by the transition to stage N1 or wake. The EMG recording of the yawning episode had morphology of a tonic episode directly linked with a phasic episode. We also observed a few episodes of sleep-talking, gasping, and loud yawning. The majority of linked yawning-bruxing episodes were present at the end of apneic/ hypopneic events followed by desaturation. Moreover, PSG outcomes revealed 23 arousals/h, including both respiratory and bruxism event-related arousals. BEI was estimated at 11.7 events/h of sleep.

#### 3.2.2 Polysomnographic examination with PAP titration

Yawning episodes were reduced but were also observed during the second vPSG performed with concomitant PAP titration, which was conducted approximately 3 weeks after the first examination. Before therapeutic intervention, the technician fitted a nasal mask, which was accepted by the patient, and set the therapeutic pressure range to 4–17 cm H2O. Analyzing the data, we have noticed the resolution of yawning episodes, to the total number estimated *n* = 8, 1.3 episodes/h, occurring mainly in N1 and N2 sleep stages. We have also noticed AHI and BEI reductions, according to the data revealed in Table 1. Yawning episodes had the same morphology assessed with EMG recording, supplemented by videorecording. During the interview, like after the first diagnostic PSG, the patient was not aware of having episodes of yawning or bruxing, and he also neglected orofacial pain.

**Table 1:** Polysomnographic parameters from the diagnostic and follow-up vPSG examinations.

Polysomnography parameter	The first polysomnographic examination	The second polysomnographic examination with PAP titration
Total Sleep Time (TST, min)	404.00	368.00
N1 sleep stage (% of TST)	37.40	26.60
N2 sleep stage (% of TST)	32.70	40.80
N3 sleep stage (% of TST)	15.70	17.10
REM (% of TST)	14.20	15.50
Sleep Latency (min)	11.90	13.40
REM Latency (min)	113.90	94.90
Wake After Onset Sleep (WASO, min)	89.50	64.30
Sleep Efficiency (SE, %)	79.90	82.60
Snore Percentage (%)	25.60	1.50
Arousal Index (n/h)	20.40	21.40
Apnea-Hypopnea Index (AHI, n/h)	33.90	4.90
Oxygen Desaturation Index (ODI, n/h)	34.10	9.00
Respiratory Disturbances Index (RDI, n/h)	33.90	4.90
Mean O2 Saturation (Mean SpO2, %)	91.70	92.80
Minimal O2 Saturation (Min. SpO2, %)	76.00	86.00
Time of saturation under 90% (T < 90%, %)	20.10	0.70
Mean desaturation drop (%)	7.00	4.20
Movement (in TST, %)	10.20	4.10
Mean Pulse (beats/min)	52.80	48.80
Minimum Pulse (beats/min)	43.00	42.00
Maximum Pulse (beats/min)	104.00	69.00
Bruxism Episode Index (BEI, n/h)	11.70	11.10
Phasic Bruxism (n/h)	11.6	10.60
Tonic Bruxism (n/h)	0.1	0.50
Mixed Bruxism (n/h)	0	0
Yawning (n/h)	5.00	1.30
Telemetric system: Mean PAP (cm H2O)	n/a	5.6
Telemetric system: Maximum PAP (cm H2O)	n/a	17.00
Telemetric system: Minimum PAP (cm H2O)	n/a	4.00
Average 95th percentile pressure (cm H2O)	n/a	8.80

Table presents a comparison between the patient’s baseline (pre-treatment) and follow-up (under autoPAP therapy) sleep study parameters. The follow-up vPSG demonstrates clinical improvement in key respiratory parameters, including apnea–hypopnea index (AHI), improvement in mean and minimum oxygen saturation, and decreased total time with oxygen saturation below 90%. Notably, the number of yawning episodes per hour also decreased in parallel with improved nocturnal oxygenation, supporting a potential pathophysiological link between desaturation events and sleep-related yawning.

### 3.3 Therapeutic intervention

After in-lab PAP titration, therapeutic intervention included positive airway pressure therapy (PAP), administered every night at the patient’s home. PAP therapy parameters included a pressure range of 4–12 cm H2O, based on a titration study. The patient was also given a prescription for an individually fitted nasal mask. The recommendations on PAP therapy included using an autoPAP device for a minimum of 4 h/night, optimally for the whole night and every night.

### 3.4 Follow-up and outcomes

After several months, the patient was admitted to the outpatient clinic to assess PAP therapy results. According to the medical interview, the tolerability and adherence to the therapy were poor. The assessed period of PAP therapy ranged from 26 April 2023 to 16 July 2023. After this time, the patient did not use autoPAP. Device settings involved a minimum pressure of 4 cm H2O, a maximum pressure of 16 cm H2O, and a median pressure of 7 cm H2O. According to the therapy compliance, the patient used the device on average for 3 h 14 min per night, for 66% of the nights. The estimated mean AHI was 5 episodes/h, and the median leakage was 2.5 L/min. Despite satisfactory therapy results, the patient was not planning to continue PAP in the future. Although he was proposed to undergo PSG hospitalization once again, he refused to do it.

## 4 Discussion

Our case report presents an unusual case of yawning during sleep with a temporal, but not causal, relationship with episodes of sleep apnea and bruxism. It is worth noting that the instrumental approach involved polysomnographic examination supplemented by video- and audio recordings performed twice in the same patient in a small-time interval before and after the therapeutic intervention with PAP.

Although yawning during sleep is an unusual presentation, alternative causes such as nocturnal epileptic seizures, brainstem lesions, or medication-related effects were considered unlikely. The patient did not report daytime confusion or any abnormal movements, and there were no focal neurological symptoms in the physical examination that would indicate central nervous system pathology. Additionally, he was not on medications known to influence dopaminergic or cholinergic pathways. Given the absence of neurological or pharmacological risk factors, further neuroimaging was not deemed necessary.

As mentioned before, several theories on yawning have been presented to date. The most crucial concept of yawning’s role assumes that this is a thermoregulatory process leading to brain cooling (13). For instance, Gallup et al. reported two consecutive cases of women reporting uncontrolled yawning attacks accompanied by body temperature lowering (14). The cooling mechanism was presented as the circulatory dynamic of peripheral and cranial blood flow. The blood flow variability was previously linked with episodes of SB as a result of cardiac activity controlled by the autonomic nervous system. Thus, the yawning episodes linked with SB episodes observed in the current case may confirm this temperature-lowering mechanism.

Second, the arousal reflex of the brain (15) against hypoxia may be linked with episodes of sleep bruxism. Micro-arousals occurring during sleep may be related to episodes of sleep bruxism, mostly observed during sleep stage transitions; thus, in our unusual case report, concomitant yawning may also contribute to this arousal mechanism, as the behavior is linked with maintaining airway patency.

This is in accordance with the theory of Doelman and Rijken (16), indicating that yawning may be a protective maneuver in preserving the airway lumen and securing long-term oxygenation, especially in individuals with a collapsible or obstructing airway, such as in OSA patients. This hypothesis may be further strengthened by several observational studies performed on patients undergoing surgeries. In these reports, it was noted that upper airway collapse during induction of anesthesia was associated with increased yawning in more than half of the patients (17, 18).

The analysis of the first diagnostic PSG found evidence for a higher number of yawning episodes, intermitting apneic events, followed by desaturation and its resolution under PAP titration. Thus, the hypothesis on yawning as a mechanism against hypoxia and arousal stimulus is also a good starting point for discussion and further research.

Contagious yawning is also considered a social behavior (3). Most of the theories focused on empathic basis of contagious yawning observed in humans and animals. Yawning occurring during sleep, which is defined as suspended consciousness of surroundings, leaves this theory unverifiable, and several questions on the socioemotional characteristics of yawning remain unanswered.

Neurochemical substrates of yawning were previously determined: dopamine, serotonin, acetylcholine, and oxytocin were previously highlighted in the literature (4). Hypodopaminergic activity was also previously discussed in the literature as bruxism-provoking factor (19). Hence, according to a similar neurochemical basis, the current temporal relationship between bruxing events and yawning may suggest the need for future investigations of dopaminergic activity in this context.

The main advantage of this case report are that, according to the methodology section, the instrumental approach to OSA diagnosis in that case involved full-night PSG supplemented by video-recording. Recordings allowed for to diagnosis of the unusual presence of yawning during sleep and obtained the most robust results in conjunction with EEG and EMG analysis. While a full polysomnographic examination with EEG and EMG remains the gold standard, in real-world settings, the use of full-night PSG may be limited by cost, access, and patient compliance. Alternative tools such as ambulatory EMG and ECG-based monitoring have gained attention as practical options for assessing SB, particularly in patients with comorbid conditions such as OSA, showing high sensitivity and specificity (20). Nevertheless, a recent study by Cid-Verdejo et al. (21) compared the diagnostic accuracy of such portable devices to PSG in patients with OSA and highlighted their limitations in clinical validation, especially in patients with moderate and severe OSA. These findings show the need for careful consideration of methodology for accurately distinguishing true bruxism activity from other motor phenomena during sleep.

The main concern about the findings of the current case report and patient perspective is that the patient did not accept CPAP treatment for a longer period. At the first evaluation, the patient did not perceive the yawning episodes during sleep and was unaware of their occurrence. The OSA diagnosis was discussed, and the patient agreed to the proposed treatment. Intervention adherence and tolerability were assessed during outpatient PAP control 7 months after diagnosis. Despite satisfying results of PAP titration, a well-fitted mask, and low leakage, the patient reported discomfort with the mask, perceived frequent air leaks, and oral dryness. His adherence was suboptimal—he used the device only 66% of nights and typically for short periods. Despite extensive counseling during the follow-up visit, he chose to discontinue therapy and declined further intervention. The patient did not attend subsequent follow-up appointments. This may support the observation that, beyond the technical aspects of diagnosis, it is also important to recognize the biopsychosocial context of sleep-related phenomena such as SB. Shared psychological and behavioral traits have been observed in patients suffering from overlapping conditions such as temporomandibular myalgia and migraine, suggesting that central sensitization, stress, and emotional regulation may contribute to the manifestation and persistence of orofacial pain and sleep disturbances (22). Although our patient did not report pain, the presence of bruxism and low adherence to therapy raises important questions about the underlying behavioral factors. In summary, the current paper describes an unusual case of concomitant behaviors: sleep bruxism and yawning during sleep in patient with diagnosed OSA. The presented case report leads to the following conclusions: first, yawning episodes were temporally linked with desaturation, as were sleep bruxism events, suggesting a secondary to hypoxia attribute of both conditions. Second, the resolution of yawning after PAP therapy strongly suggests a hypoxic basis for yawning. Based on the literature, both conditions are defined as complex issues requiring future research.

## Data Availability

The original contributions presented in the study are included in the article/supplementary material, further inquiries can be directed to the corresponding author.
